# On *θ*_*ω*_ continuity

**DOI:** 10.1016/j.heliyon.2020.e03349

**Published:** 2020-02-05

**Authors:** Samer Al Ghour, Bayan Irshidat

**Affiliations:** Department of Mathematics and Statistics, Jordan University of Science and Technology, Irbid 22110, Jordan

**Keywords:** Mathematics, *θ*-open, *ω*-open, $\theta_{\omega }$-open, *θ*-continuous, Weakly continuous, Faintly continuous

## Abstract

We use the closure and the theta omega closure operators to introduce $\theta_{\omega }$-continuous, *ω*-*θ*-continuous, weakly $\theta_{\omega }$-continuous and faintly $\theta_{\omega }$-continuous as new four classes of functions. We obtain several properties, relationships, examples and counter-examples related to them.

## 1 Introduction and preliminaries

Let *A* be any subset of a topological space (X,τ). The set {x∈X:∀O∈τ with x∈O, O∩Ais uncountable} is called the set of condensation points of *A* and is denoted by Cond(A). *A* is called an *ω*-closed set [1] if Cond(A)⊆A and *A* is called an *ω*-open set if X−A is *ω*-closed. The family of all *ω*-open sets of (X,τ) forms a topology on *X* which is finer than *τ* and this topology is denoted by $\tau_{\omega }$. *ω*-open sets played a vital role in general topology research see, [2], [3], [4], [5], [6], [7], [8], [9]. The closure of *A* in (X,τ) (resp. $\left(X,\tau_{\omega }\right)$) will be denoted by $\bar{A}$ (resp. ${\bar{A}}^{\omega }$). $Cl_{\theta }\left(A\right)=\left\{x\in X:\forall O\in \tau \text{ with }x\in O\text{, }\bar{O}\cap A\neq \emptyset \right\}$ [10] and $Cl_{\theta_{\omega }}\left(A\right)=\left\{x\in X:\forall O\in \tau \text{ with }x\in O\text{, }{\bar{O}}^{\omega }\cap A\neq \emptyset \right\}$ [11] are the *θ*-closure and the $\theta_{\omega }$-closure operators. *A* is *θ*-closed [10] (resp. $\theta_{\omega }$-closed [11]) if $Cl_{\theta }\left(A\right)=A$ (resp. $Cl_{\theta_{\omega }}\left(A\right)=A$). *A* is *θ*-open [10] (resp. $\theta_{\omega }$-open [11]) if X−A is *θ*-closed (resp. $\theta_{\omega }$-closed). The family of *θ*-open (resp. $\theta_{\omega }$-open) sets in (X,τ) are denoted by $\tau_{\theta }$ (resp. $\tau_{\theta_{\omega }}$). It is proved in [10] and [11] that $\left(X,\tau_{\theta }\right)$ and $\left(X,\tau_{\theta_{\omega }}\right)$ are topological spaces, $\tau_{\theta }$ coarser than $\tau_{\theta_{\omega }}$ and $\tau_{\theta_{\omega }}$ coarser than *τ*. Authors in [11] defined and investigated $\theta_{\omega }$-open sets. And they used them to characterize some separation axioms. Studying continuities in topological spaces is still a hot area of research see, [12], [13], [14], [15], [16]. This paper is devoted to introduce and investigate four new classes of functions, namely: $\theta_{\omega }$-continuous, *ω*-*θ*-continuous, weakly $\theta_{\omega }$-continuous and faintly $\theta_{\omega }$-continuous.

In this paper, for any nonempty set *X*, $\tau_{ind},\tau_{cof},\tau_{coc}$ will denote respectively the indiscrete topology, the cofinite topology, and the cocountable topology on *X*.

The following sequence of definitions and propositions will be used in the sequel:

Definition 1.1 [17] A topological space (X,τ) is called anti-locally countable if each U∈τ−{∅} is uncountable.

Proposition 1.2 *[18]* *If* (X,τ) *is an anti-locally countable topological space, then for all* $A\in \tau_{\omega },{\bar{A}}^{\omega }=\bar{A}$*.*

Definition 1.3 [18] A topological space (X,τ) is called *ω*-regular if for each closed set *F* in (X,τ) and x∈X−F, there exist U∈τ and $V\in \tau_{\omega }$ such that x∈U,F⊆V and U∩V=∅.

Proposition 1.4 *[18]* *A topological space* (X,τ) *is ω-regular if and only if for each* U∈τ *and each* x∈U *there is* V∈τ *such that* $x\in V\subseteq {\bar{V}}^{\omega }\subseteq U$*.*

Recall that a topological space (X,τ) is locally indiscrete if every open set in (X,τ) is closed and (X,τ) is locally countable if for each x∈X, there is U∈τ such that x∈U and *U* is countable.

Definition 1.5 [11] A topological space (X,τ) is said to be *ω*-locally indiscrete if every open set in (X,τ) is *ω*-closed.

Proposition 1.6 *[11]* *a. Every locally indiscrete topological space is ω-locally indiscrete.*

*b. Every locally countable topological space is ω-locally indiscrete.*

## 2 Continuity

Definition 2.1 [19] A function f:(X,τ)⟶(Y,σ) is said to be *θ*-continuous if for every x∈X and every open subset *V* in *Y* containing f(x), there exists an open subset *U* in *X* containing *x* such that $f(\bar{U})\subseteq \bar{V}$.

Definition 2.2 A function f:(X,τ)⟶(Y,σ) is said to be $\theta_{\omega }$-continuous if for every x∈X and every open subset *V* in *Y* containing f(x), there exists an open subset *U* in *X* containing *x* such that $f(\bar{U})\subseteq {\bar{V}}^{\omega }$.

Theorem 2.3 *Every* $\theta_{\omega }$*-continuous function is θ-continuous.*

Proof Let f:(X,τ)⟶(Y,σ) be $\theta_{\omega }$-continuous. Let x∈X and let *V* be any open set in *Y* containing f(x). Since *f* is $\theta_{\omega }$-continuous, there exists an open subset *U* in *X* containing *x* such that $f(\bar{U})\subseteq {\bar{V}}^{\omega }\subseteq \bar{V}$. It follows that *f* is *θ*-continuous. 

The converse of Theorem 2.3 is not true in general as the following example clarifies:

Example 2.4 Consider the function $f:\left(N,\tau_{ind}\right)⟶\left(N,\tau_{cof}\right)$ defined as f(x)=x. Then

a. *f* is *θ*-continuous.

b. *f* is not $\theta_{\omega }$-continuous.

Proof a. Let x∈N and let $V\in \tau_{cof}$ such that f(x)=x∈V. Take U=N. Then $x\in U\in \tau_{ind}$ and $f\left(\bar{U}\right)\subseteq \bar{V}$ =N. It follows that *f* is *θ*-continuous.

b. Let x=1 and let V=N−{2}. Then $V\in \tau_{cof}$ with f(1)=1∈V. If there exists $U\in \tau_{ind}$ such that $1\in U\in \tau_{ind}$ and $f\left(\bar{U}\right)\subseteq {\bar{V}}^{\omega }$, then U=N and f(N)=N. But ${\bar{V}}^{\omega }=N-\{2\}$. It follows that *f* is not $\theta_{\omega }$-continuous. 

Theorem 2.5 *If* f:(X,τ)⟶(Y,σ) *is a θ-continuous function and* (Y,σ) *is an anti-locally countable topological space, then* f:(X,τ)⟶(Y,σ) *is* $\theta_{\omega }$*-continuous.*

Proof Let x∈X and let *V* be any open subset in *Y* containing f(x). Since *f* is *θ*-continuous, there exists an open subset *U* in *X* containing *x* such that $f(\bar{U})\subseteq \bar{V}$. Since (Y,σ) is anti-locally countable, then by Proposition 1.2 we have $\bar{V}={\bar{V}}^{\omega }$ and thus $f(\bar{U})\subseteq {\bar{V}}^{\omega }$. It follows that *f* is $\theta_{\omega }$-continuous. 

Theorem 2.6 *[19]* *Every continuous function is θ-continuous but not conversely.*

The following two examples show that continuity and $\theta_{\omega }$-continuity are independent:

Example 2.7 Consider the function $f:\left(R,\tau_{ind}\right)⟶\left(R,\tau_{cof}\right)$ defined as f(x)=x. Clearly that *f* is discontinuous. Let x∈R and let $V\in \tau_{cof}$ such that f(x)=x∈V. Then ${\bar{V}}^{\omega }=R$. Take U=R. Then $x\in U\in \tau_{ind}$ and $f\left(\bar{U}\right)=R\subseteq R={\bar{V}}^{\omega }$. It follows that *f* is $\theta_{\omega }$-continuous.

Example 2.8 Consider the function f:(N,τ)⟶(N,σ) where τ=σ={∅,N,{1}} and f(x)=x. Clearly that *f* is continuous. Suppose that *f* is $\theta_{\omega }$-continuous. Take x=1 and V={1}. Then V∈σ with f(x)=x∈V and ${\bar{V}}^{\omega }=V$. On the other hand, if U∈τ with 1∈U, then either U={1} or U=N. In both cases, $\bar{U}=N$ and $f\left(\bar{U}\right)=N\subseteq {\bar{V}}^{\omega }=\left\{1\right\}$ which is impossible. It follows that *f* is not $\theta_{\omega }$-continuous.

In the following result we give a sufficient condition for a $\theta_{\omega }$-continuous function to be continuous:

Theorem 2.9 *If* f:(X,τ)⟶(Y,σ) *is a* $\theta_{\omega }$*-continuous function with* (Y,σ) *is ω-regular, then f is continuous.*

Proof Let x∈X and let *V* be any open set in *Y* containing f(x). Since (Y,σ) is *ω*-regular, then by Proposition 1.4 there exists an open set *H* in *Y* such that $f\left(x\right)\in H\subseteq {\bar{H}}^{\omega }\subseteq V$. Since *f* is $\theta_{\omega }$-continuous, there exists an open set *U* in *X* containing *x* such that $f(\bar{U})\subseteq {\bar{H}}^{\omega }$. Thus we have

$$f(U)\subseteq f(\bar{U})\subseteq {\bar{H}}^{\omega }\subseteq V\text{.}$$

 

It follows that *f* is continuous.

Definition 2.10 [20] A function f:(X,τ)⟶(Y,σ) is said to be weakly continuous if for every x∈X and every open set *V* in *Y* containing f(x), there exists an open subset *U* in *X* containing *x* such that $f(U)\subseteq \bar{V}$.

Definition 2.11 A function f:(X,τ)⟶(Y,σ) is said to be *ω*-*θ*-continuous if for every x∈X and every open set *V* in *Y* containing f(x), there exists an open subset *U* in *X* containing *x* such that $f({\bar{U}}^{\omega })\subseteq \bar{V}$.

Theorem 2.12 *Every ω-θ-continuous function is weakly continuous.*

Proof Let f:(X,τ)⟶(Y,σ) be *ω*-*θ*-continuous. Let x∈X and let *V* be any open set in *Y* containing f(x). Since *f* is *ω*-*θ*-continuous, there exists an open set *U* in *X* containing *x* such that $f({\bar{U}}^{\omega })\subseteq \bar{V}$. Thus $f(U)\subseteq f({\bar{U}}^{\omega })\subseteq \bar{V}$. It follows that *f* is weakly continuous. 

Theorem 2.13 *If* f:(X,τ)⟶(Y,σ) *is weakly continuous such that* (X,τ) *is ω-locally indiscrete, then f is ω-θ-continuous.*

Proof Let x∈X and let *V* be any open set in *Y* containing f(x). Since *f* is weakly continuous, there exists an open set *U* in *X* containing *x* such that $f(U)\subseteq \bar{V}$. Since (X,τ) is *ω*-locally indiscrete, then *U* is *ω*-closed and ${\bar{U}}^{\omega }=U$. Thus $f({\bar{U}}^{\omega })=f\left(U\right)\subseteq \bar{V}$. It follows that *f* is *ω*-*θ*-continuous. 

Corollary 2.14 *If* f:(X,τ)⟶(Y,σ) *is weakly continuous such that* (X,τ) *is locally indiscrete, then f is ω-θ-continuous.*

Proof Proposition 1.6 (a) and Theorem 2.13. 

Corollary 2.15 *If* f:(X,τ)⟶(Y,σ) *is weakly continuous such that* (X,τ) *is locally countable, then f is ω-θ-continuous.*

Proof Proposition 1.6 (b) and Theorem 2.13. 

Theorem 2.16 *If* f:(X,τ)⟶(Y,σ) *is weakly continuous such that* (X,τ) *is ω-regular, then f is ω-θ-continuous.*

Proof Let x∈X and let *V* be any open set in *Y* containing f(x). Since *f* is weakly continuous, there exists an open set *H* in *X* containing *x* such that $f(H)\subseteq \bar{V}$. Since (X,τ) is *ω*-regular, then there is an open set *U* in *X* containing *x* such that ${\bar{U}}^{\omega }\subseteq H$. Thus $f({\bar{U}}^{\omega })=f\left(H\right)\subseteq \bar{V}$. It follows that *f* is *ω*-*θ*-continuous. 

Theorem 2.17 *Every θ-continuous function is ω-θ-continuous.*

Proof Let f:(X,τ)⟶(Y,σ) be *θ*-continuous. Let x∈X and let *V* be any open set in *Y* containing f(x). Since *f* is *θ*-continuous, there exists an open set *U* in *X* containing *x* such that $f(\bar{U})\subseteq \bar{V}$. Thus $f({\bar{U}}^{\omega })\subseteq f(\bar{U})\subseteq \bar{V}$. It follows that *f* is *ω*-*θ*-continuous. 

Theorem 2.18 *If* f:(X,τ)⟶(Y,σ) *is ω-θ-continuous such that* (X,τ) *is anti-locally countable, then f is θ-continuous.*

Proof Let x∈X and let *V* be any open set in *Y* containing f(x). Since *f* is *ω*-*θ*-continuous, there exists an open set *U* in *X* containing *x* such that $f({\bar{U}}^{\omega })\subseteq \bar{V}$. Since (X,τ) is anti-locally countable, then by Proposition 1.4, ${\bar{U}}^{\omega }=\bar{U}$. Thus $f(\bar{U})=f\left({\bar{U}}^{\omega }\right)\subseteq \bar{V}$. It follows that *f* is *θ*-continuous. 

Remark 2.19 The function in Example 3.3 of [21] is weakly continuous but not *θ*-continuous, moreover, its domain is anti-locally countable. So by Theorem 2.18, this function is not *ω*-*θ*-continuous. Therefore, the converse of Theorem 2.12 is not true in general.

The following example shows that the converse of Theorem 2.17 is not true in general:

Example 2.20 We utilize Example 3.2 of [21]. Let X=Y={a,b,c,d} and τ=σ={∅,X,{b},{c},{b,c},{a,b},{a,b,c},{b,c,d}}. Define f:(X,τ)⟶(Y,σ) by f(a)=c,f(b)=d,f(c)=b,f(d)=a. As appear in [21] *f* is weakly continuous and by Corollary 2.15 *f* is *ω*-*θ*-continuous. On the other hand, it is proved in [21] that *f* is not *θ*-continuous.

Definition 2.21 A function f:(X,τ)⟶(Y,σ) is said to be weakly $\theta_{\omega }$-continuous if for every x∈X and every open set *V* in *Y* containing f(x), there exists an open subset *U* in *X* containing *x* such that $f(U)\subseteq {\bar{V}}^{\omega }$.

Theorem 2.22 *Every weakly* $\theta_{\omega }$*-continuous function is weakly continuous.*

Proof Let f:(X,τ)⟶(Y,σ) be weakly $\theta_{\omega }$-continuous. Let x∈X and let *V* be any open set in *Y* containing f(x). Since *f* is weakly $\theta_{\omega }$-continuous, there exists an open set *U* in *X* containing *x* such that $f(U)\subseteq {\bar{V}}^{\omega }$. Thus $f(U)\subseteq {\bar{V}}^{\omega }\subseteq \bar{V}$. It follows that *f* is weakly continuous. 

The following example shows that the converse of Theorem 2.22 is not true in general:

Example 2.23 Consider the identity function f:(N,τ)⟶(N,σ) where τ={∅,N} and σ={∅,N,{1}}. It is not difficult to check that $\bar{\left\{1\right\}}=N$ and ${\bar{\left\{1\right\}}}^{\omega }=\left\{1\right\}$. To see that *f* is weakly continuous, let x∈N and V∈σ such that f(x)=x∈V. Then V=N or V={1} and in both cases $\bar{V}=N$. Choose U=N. Then x∈U∈τ and $f\left(U\right)=N\subseteq N=\bar{V}$. To see that *f* is not weakly $\theta_{\omega }$-continuous, suppose to the contrary that *f* is weakly $\theta_{\omega }$-continuous. Let x=1 and take V={1}. Then there is U∈τ such that 1∈U and $f\left(U\right)\subseteq {\bar{\left\{1\right\}}}^{\omega }$. Then $f\left(U\right)=f\left(N\right)=N\subseteq {\bar{\left\{1\right\}}}^{\omega }=\left\{1\right\}$ which is a contradiction.

Theorem 2.24 *If* f:(X,τ)⟶(Y,σ) *is weakly continuous such that* (Y,σ) *is anti-locally countable, then f is weakly* $\theta_{\omega }$*-continuous.*

Proof Let x∈X and let *V* be any open set in *Y* containing f(x). Since *f* is weakly continuous, there exists an open set *U* in *X* containing *x* such that $f(U)\subseteq \bar{V}$. Since (Y,σ) is anti-locally countable, then by Proposition 1.4, ${\bar{V}}^{\omega }=\bar{V}$, and so $f(U)\subseteq \bar{V}={\bar{V}}^{\omega }$. It follows that *f* is weakly $\theta_{\omega }$-continuous. 

Theorem 2.25 *Every continuous function is weakly* $\theta_{\omega }$*-continuous.*

Proof Let f:(X,τ)⟶(Y,σ) be continuous. Let x∈X and let *V* be any open set in *Y* containing f(x). Since *f* is continuous, there exists an open set *U* in *X* containing *x* such that f(U)⊆V. Thus $f(U)\subseteq V\subseteq {\bar{V}}^{\omega }$. It follows that *f* is weakly $\theta_{\omega }$-continuous. 

The following example shows that the converse of Theorem 2.25 is not true in general:

Example 2.26 Consider the identity function f:(R,τ)⟶(R,σ) where $\tau =\tau_{u}$ and $\sigma =\tau_{coc}$. Clearly that *f* is discontinuous. Note $\sigma_{\omega }=\sigma =\tau_{coc}$. Let x∈R and let V∈σ such that f(x)=x∈V. Then ${\bar{V}}^{\omega }=R$. Take U=R. Then x∈U∈τ and $f\left(U\right)=R\subseteq R={\bar{V}}^{\omega }$. It follows that *f* is weakly $\theta_{\omega }$-continuous.

Theorem 2.27 *If* f:(X,τ)⟶(Y,σ) *is weakly* $\theta_{\omega }$*-continuous such that* (Y,σ) *is ω-locally indiscrete, then f is continuous.*

Proof Let x∈X and let *V* be any open set in *Y* containing f(x). Since *f* is weakly $\theta_{\omega }$-continuous, there exists an open set *U* in *X* containing *x* such that $f(U)\subseteq {\bar{V}}^{\omega }$. Since (Y,σ) is *ω*-locally indiscrete, then *V* is *ω*-closed and ${\bar{V}}^{\omega }=V$. Thus $f(U)\subseteq {\bar{V}}^{\omega }=V$. It follows that *f* is continuous. 

Corollary 2.28 *If* f:(X,τ)⟶(Y,σ) *is weakly* $\theta_{\omega }$*-continuous such that* (Y,σ) *is locally indiscrete, then f is continuous.*

Proof Proposition 1.6 (a) and Theorem 2.27. 

Corollary 2.29 *If* f:(X,τ)⟶(Y,σ) *is weakly* $\theta_{\omega }$*-continuous such that* (Y,σ) *is locally countable, then f is continuous.*

Proof Proposition 1.6 (b) and Theorem 2.27. 

Theorem 2.30 *Every* $\theta_{\omega }$*-continuous function is weakly* $\theta_{\omega }$*-continuous.*

Proof Let f:(X,τ)⟶(Y,σ) be $\theta_{\omega }$-continuous. Let x∈X and let *V* be any open set in *Y* containing f(x). Since *f* is $\theta_{\omega }$-continuous, there exists an open set *U* in *X* containing *x* such that $f(\bar{U})\subseteq {\bar{V}}^{\omega }$. Thus $f(U)\subseteq f(\bar{U})\subseteq {\bar{V}}^{\omega }$. It follows that *f* is weakly $\theta_{\omega }$-continuous. 

The following example shows that the converse of Theorem 2.30 is not true in general:

Example 2.31 Take *f* as in Example 2.8. Since *f* is continuous, then by Theorem 2.25, it is weakly $\theta_{\omega }$-continuous. On the other hand, it is proved in Example 2.8 that *f* is not $\theta_{\omega }$-continuous.

Definition 2.32 [22] A function f:(X,τ)⟶(Y,σ) is said to be faintly continuous if for every x∈X and every *θ*-open set *V* in *Y* containing f(x), there exists an open subset *U* in *X* containing *x* such that f(U)⊆V.

Definition 2.33 A function f:(X,τ)⟶(Y,σ) is said to be faintly $\theta_{\omega }$-continuous if for every x∈X and every $\theta_{\omega }$-open set *V* in *Y* containing f(x), there exists an open subset *U* in *X* containing *x* such that f(U)⊆V.

Theorem 2.34 *[22]* *Continuity* ⟹ *weak continuity* ⟹ *faint continuity.*

Theorem 2.35 *[22]* *Let* f:(X,τ)⟶(Y,σ) *be a function. Then the following are equivalent:*

*a.* f:(X,τ)⟶(Y,σ) *is faintly θ-continuous.*

*b.* $f:\left(X,\tau \right)⟶\left(Y,\sigma_{\theta }\right)$ *is continuous.*

The following characterizations of faintly $\theta_{\omega }$-continuity follow directly:

Theorem 2.36 *Let* f:(X,τ)⟶(Y,σ) *be a function. Then the following are equivalent:*

*a.* f:(X,τ)⟶(Y,σ) *is faintly* $\theta_{\omega }$*-continuous.*

*b.* $f:\left(X,\tau \right)⟶\left(Y,\sigma_{\theta_{\omega }}\right)$ *is continuous.*

*c. The inverse image of each* $\theta_{\omega }$*-open set in Y is open in X.*

*d. The inverse image of each* $\theta_{\omega }$*-closed set in Y is closed in X.*

Theorem 2.37 *Every weakly* $\theta_{\omega }$*-continuous function is faintly* $\theta_{\omega }$*-continuous.*

Proof Let f:(X,τ)⟶(Y,σ) be weakly $\theta_{\omega }$-continuous. Let x∈X and *V* be $\theta_{\omega }$-open set containing f(x). There is B∈σ such that $f\left(x\right)\in B\subseteq {\bar{B}}^{\omega }\subseteq V$. Since *f* is weakly $\theta_{\omega }$-continuous, then there exists U∈τ containing *x* such that $f\left(U\right)\subseteq {\bar{B}}^{\omega }\subseteq V$. It follows that *f* is faintly $\theta_{\omega }$-continuous. 

The following example shows that the implication in Theorem 2.37 is not reversible in general:

Example 2.38 Consider the identity function f:(R,σ)⟶(R,τ) where σ={∅,R,N} and *τ* as in Example 2.26. Then

a. *f* is faintly $\theta_{\omega }$-continuous.

b. *f* is not weakly continuous.

Proof (a) Since by Example 2.26 $\tau_{\theta_{\omega }}=\left\{\emptyset ,R,N\right\}=\sigma$, then $f:\left(R,\sigma \right)⟶\left(R,\tau_{\theta_{\omega }}\right)$ is continuous and by Theorem 2.36 f:(R,σ)⟶(R,τ) is faintly $\theta_{\omega }$-continuous.

(b) Suppose to the contrary that *f* is weakly continuous. Since $f\left(\sqrt{2}\right)=\sqrt{2}\in Q^{c}\in \tau$, then there is U∈σ such that $\sqrt{2}\in U$ and $f\left(U\right)\subseteq \bar{Q^{c}}=R-N$. Since $\sqrt{2}\in U\in \sigma$, then U=R and f(U)=R, a contradiction. 

Theorem 2.39 *Every faintly* $\theta_{\omega }$*-continuous function is faintly continuous.*

Proof Theorem 2.18, Theorem 2.35, Theorem 2.36. 

The implication in Theorem 2.39 is not reversible as it can be seen from the following example:

Example 2.40 Consider the identity function f:(R,τ)⟶(R,σ) where τ={∅,R} and σ={∅,R,Q}. It is not difficult to check that $\sigma_{\theta }=\left\{\emptyset ,R\right\}$ and $\sigma_{\theta_{\omega }}=\sigma$. Therefore, by Theorem 2.35, Theorem 2.36, it follows that *f* is faintly continuous but not faintly $\theta_{\omega }$-continuous.

We can summarize the results and examples above by means of the following diagram:

**Figure:**
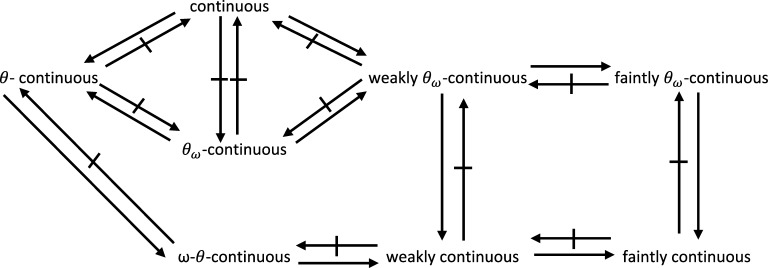

## Declarations

### Author contribution statement

S. Al Ghour, B. Irshidat: Conceived and designed the experiments; Performed the experiments; Analyzed and interpreted the data; Contributed reagents, materials, analysis tools or data; Wrote the paper.

### Funding statement

This research did not receive any specific grant from funding agencies in the public, commercial, or not-for-profit sectors.

### Competing interest statement

The authors declare no conflict of interest.

### Additional information

No additional information is available for this paper.
